# *In vivo *patch-clamp analysis of response properties of rat primary somatosensory cortical neurons responding to noxious stimulation of the facial skin

**DOI:** 10.1186/1744-8069-6-30

**Published:** 2010-05-26

**Authors:** Mamoru Takeda, Masayuki Takahashi, Masanori Nasu, Shigeji Matsumoto

**Affiliations:** 1 Department of Physiology, School of Life Dentistry at Tokyo, Nippon Dental University, 1-9-20, Fujimi-cho, Chiyoda-ku, Tokyo, 102-8159, Japan; 2 Research Center for Odontology, School of Life Dentistry at Tokyo, Nippon Dental University, 1-9-20, Fujimi-cho, Chiyoda-ku, Tokyo, 102-8159, Japan

## Abstract

**Background:**

Although it has been widely accepted that the primary somatosensory (SI) cortex plays an important role in pain perception, it still remains unclear how the nociceptive mechanisms of synaptic transmission occur at the single neuron level. The aim of the present study was to examine whether noxious stimulation applied to the orofacial area evokes the synaptic response of SI neurons in urethane-anesthetized rats using an *in vivo *patch-clamp technique.

**Results:**

*In vivo *whole-cell current-clamp recordings were performed in rat SI neurons (layers III-IV). Twenty-seven out of 63 neurons were identified in the mechanical receptive field of the orofacial area (36 neurons showed no receptive field) and they were classified as non-nociceptive (low-threshold mechanoreceptive; 6/27, 22%) and nociceptive neurons. Nociceptive neurons were further divided into wide-dynamic range neurons (3/27, 11%) and nociceptive-specific neurons (18/27, 67%). In the majority of these neurons, a proportion of the excitatory postsynaptic potentials (EPSPs) reached the threshold, and then generated random discharges of action potentials. Noxious mechanical stimuli applied to the receptive field elicited a discharge of action potentials on the barrage of EPSPs. In the case of noxious chemical stimulation applied as mustard oil to the orofacial area, the membrane potential shifted depolarization and the rate of spontaneous discharges gradually increased as did the noxious pinch-evoked discharge rates, which were usually associated with potentiated EPSP amplitudes.

**Conclusions:**

The present study provides evidence that SI neurons in deep layers III-V respond to the temporal summation of EPSPs due to noxious mechanical and chemical stimulation applied to the orofacial area and that these neurons may contribute to the processing of nociceptive information, including hyperalgesia.

## Background

Many studies have reported that multiple cortical areas, including the primary somatosensory (SI) cortex, are involved in nociception [1-3]. The processing of the SI cortex has been analysed by non-invasive imaging techniques such as magnetoencephalography, positron emission tomography and functional magnetic resonance imaging, and the human SI cortex responds to noxious stimuli [4]. Extracellular unit recording techniques have demonstrated that monkey and cat SI neurons in the deeper lamina encode the intensity of noxious mechanical, thermal, chemical stimulation [5-9], and that rat SI cortical neurons respond to noxious mechanical stimulation applied to the trigeminal receptive field and these neurons are classified as nociceptive-specific (NS) or wide-dynamic range (WDR) neurons (either noxious or non-noxious stimulation-responding) [10-12]. Although it has been accepted that the SI neuron plays an important role in the sensory discriminative aspect of pain perception [6,9], the mechanism of pain processing in the SI neuron is still unknown, unlike tactile-sensory processing. In particular, imaging and extracellular recording methods are not applicable for revealing the mechanisms of synaptic transmission at the single neuron level.

Transient receptor potential ankyrin 1 (TRPA1) is a member of the TRP superfamily of ion channel proteins which have been implicated in thermo-, chemo- and mechano-sensation [13-17]. It has recently been demonstrated that inhibition of TRPA1 function reduces mechanical hypersensitivity produced by inflammation [15,17]. The chemical irritant, mustard oil (MO), is an agonist of TRPA1 [13,18] and has long been known to activate somatosensory neurons, resulting in acute pain and neurogenic inflammation through peripheral release of neuropeptides from the primary afferent nerve terminal [19]. Concerning superficial spinal dorsal horn neurons in the first relay station of the pain pathway, it has been reported that peripheral application of MO produces a prolonged increase in the responses to high-intensity mechanical stimulus applied to the receptive field, and this effect is characterized by a depolarization response as well as an increased amplitude of the excitatory postsynaptic potentials (EPSPs) evoked by mechanical stimulation [20]. There is evidence that the application of MO into several orofacial tissues induces hyperexcitability of the trigeminal spinal nucleus caudalis neurons, corresponding with trigeminal hyperalgesia [21-24]. A previous study has clearly demonstrated that a significantly increased pinch-evoked responsiveness of SI neurons occurs after carrageenin-induced hindpaw inflammation in a single unit recording [25]. From these observations, we hypothesized that SI neurons may contribute to encoding the noxious stimuli applied to the orofacial area in accordance with the temporal summation of EPSPs. To date, however, no studies have been conducted to test this hypothesis

The aim of the present study, therefore, was to examine whether noxious mechanical and chemical stimulation applied to the orofacial area modulate the synaptic response of SI neurons using *in vivo *patch-clamp techniques, as modified by a previous study [26].

## Methods

Experiments performed in the present study were approved by the Animal Use and Care Committee of Nippon Dental University and were consistent with the ethical guidelines of the International Association for the Study of Pain [27]. Every effort was made to minimize the number of animals used and their suffering.

### Animal preparation

The experiments were performed on 21 male Wistar rats (250-380 g body weight). They were initially anesthetized with urethane (1.2-1.5 g/kg, ip). The animals were then placed in a stereotaxic apparatus, and a partial craniotomy was performed to expose the SI cortex (including the barrel cortex) on the right side (3-6 mm lateral to the midline, and from 0.5 mm anterior to 2.5 mm posterior to bregma), as described in previous studies [11,28]. Following removal of the cranial bone, enzymatic treatment of the dura mater was initiated, using collagenase, in order to obtain stable *in vivo *whole-cell recordings [26]. Briefly, the 37°C enzyme solution (collagenase type II and XI, 50 mg/ml, 1:1) was applied with a small piece of filter paper on top of the dura for 20-30 min. After treatment with the enzymatic solution, the area was rinsed thoroughly with warm physiological saline and the body temperature was measured with a rectal probe and maintained with a homeothermic blanket at 36.5 ± 0.5°C during recording. Under urethane anesthesia, oxygen was supplied through a nose cone as described previously [29]. Adequacy of the anesthesia was determined by the lack of a response to pinching a paw. Additional anesthesia was given when pinching the paw resulted in a withdrawal reflex [30].

### *In vivo* patch-clamp recordings from SI neurons

Whole-cell recordings were performed from the SI neurons with a patch electrode (thin-walled borosilicate glass capillary, resistance 7-12 MΩ) filled with an internal solution of the following composition (in mM): potassium gluconate, 110; KCl, 20; HEPES, 10; EGTA, 10; MgATP, 2; Na_2_ATP, 5; Na-GTP, 0.1; biocytin, 20; pH 7.3; with 100 μg/ml amphotericin B [31-33]. We conducted stable *in vivo *whole-cell current-clamp recordings, as modified by a previous study [26]. Briefly, while maintaining a positive pressure, the electrode pipette was advanced into the cortex, where close contact with a neuron was recognized by an increase in the resistance of the electrode and/or a sudden increase in the spontaneous discharge rate. At this point the positive pressure was released and a small negative pressure was applied. This often resulted in a gradual entry into the cell interior, as indicated by a slow increase in the membrane potential. After making a giga-ohm seal (> 1 GΩ), access resistance was gradually reduced by perforation (caused by amphotericin B), a brief period of negative pressure as well as using a zap input current to obtain the whole-cell configuration. All *in vivo *data were collected when the access resistance of the recording was < 50 MΩ [34]. Current-clamp recordings were conducted with an Axopatch 200B amplifier (Molecular Devices, Foster City, CA, USA). Signals were low-pass filtered at 1 or 5 kHz and digitized at 10 kHz. No significant changes were found in access resistance throughout the experiments. The input resistance was calculated by injecting negative current (50-100 pA, 250 ms) into the soma, and determined the voltage drop after current injection, as described in our previous studies [31,32]. Data were stored on a computer disk for off-line analysis. Drug effects were analyzed using one way analysis of variance, followed by Dunnett's test (post hoc test). *P *< 0.05 was considered statistically significant.

### Noxious mechanical stimulation of orofacial area

Somatic receptive fields of the SI neurons were first determined by applying non-noxious stimuli with a paintbrush (< 150 mN) and response to noxious stimulation was assessed by pinching the skin near a whisker pad with forceps (calibrated forceps at an intensity of 4.0 N, 1-3 s), a stimulus that evoked pain sensation when applied to human subjects. The size of the mechanical receptive field of neurons was identified by probing the skin with von Frey filaments, as described in our previous study [30].

### Noxious chemical stimulation

In 4 neurons, we tested whether noxious chemical stimulation by MO (allyl isothiocyanate, Sigma-Aldrich) alters noxious mechanical stimulation evoked- and spontaneous- SI neuronal activities. In order to stimulate the nociceptors in their receptive field, 10 μl of 5% MO in paraffin oil was injected into the receptive field by means of a blunt Hamilton syringe. After noxious chemical stimulation of the receptive field, we evaluated the changes in membrane potential, spontaneous, pinch-evoked discharge rates and EPSP amplitude to noxious stimulation at 5 min intervals for 40 min, since our previous study indicated the duration of significant change in the mechanical receptive field properties of upper cervical spinal dorsal horn neurons was 40 min. In a control experiment, vehicle (paraffin oil, same volume of MO) was administered to the receptive field and any significant change in SI neuronal activity was recorded for 60 min.

### Histological identification of SI neurons

The location and morphological features of recorded SI neurons were further confirmed in some instances by an intracellular injection of biocytin (2% in the electrode solution). At the end of experiments, the animal was deeply anesthetized with supplemental urethane, and perfused transcardially with heparinized saline in 0.01 M phosphate buffered saline (PBS) followed by 4% paraformaldehyde in 0.1 M phosphate buffer (pH 7.3). The cortex was removed and incubated in 4%, 10% and 20% sucrose solution for 3 × 5 min, 1 h and 2 h, respectively, and then placed in 30% sucrose for incubation overnight. The tissue was sectioned on a cryostat (Leica, Germany) at a thickness of 80-150 μm. The sections were thoroughly washed in PBS followed by Tris-buffered saline and were pretreated for 1 h in a 0.3% solution of Triton X100 in PBS. Biocytin-labeled neurons were identified by incubating the tissue overnight at 4°C in avidin-biotin-horseradish peroxidase complex, diluted 1:500 in PBS. The enzymatic reaction was revealed with diaminobenzidine (0.06%) and H_2_O_2 _(0.003%) in Tris-buffered saline for 15 min. Sections were rinsed and then mounted on silan-coated glass slides. The sections were viewed and photographed with a microscope (Leica, Germany).

## Results

### General properties of SI cortical neurons

**Figure 1:**
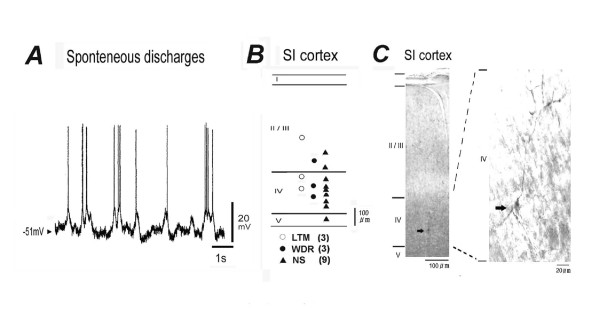
***In vivo *patch-clamp recording from rat SI neurons**. ***A***: Typical example of spontaneous discharges of SI neurons. The excitatory postsynaptic potentials (EPSPs) and inhibitory postsynaptic potentials (IPSPs) are shown by fluctuations above and below the resting membrane potential. Arrow head shows resting membrane potential. ***B***: Location of each type of SI neurons successfully identified by biocytin injection. LTM: low-threshold mechanoreceptive, WDR: wide-dynamic range, NS: nociceptive specific. Values in parentheses are the number of neurons. ***C***: Typical example of a biocytin-injected noxious specific SI neuron in layer IV with spontaneous discharges. Right panel, higher-power photomicrograph of left.

Stable whole-cell patch-clamp (current-clamp) recordings were obtained from 63 SI neurons located in the SI cortex which received sensory inputs from the contralateral whisker pad area. Most of these neurons (61/63, 97%) showed spontaneous discharges (Fig. 1A). As shown in Fig. 1A, the background activity was entirely subthreshold in some neurons, consisting of composite EPSPs. In the majolity of these neurons, a proportion of these EPSPs reached the threshold, generating random discharges of action potentials with an average frequency of 0.5-17 Hz (5.2 ± 1.9 Hz, n = 61). These SI neurons showed oscillatory property (up and down states of membrane potential at same frequency), as described in previous studies [34]. All of the SI neurons examined had membrane potentials more negative than -50 mV. The series resistance was between 20 and 50 MΩ.

### Response properties of SI neurons responding to noxious mechanical stimulation

**Figure 2:**
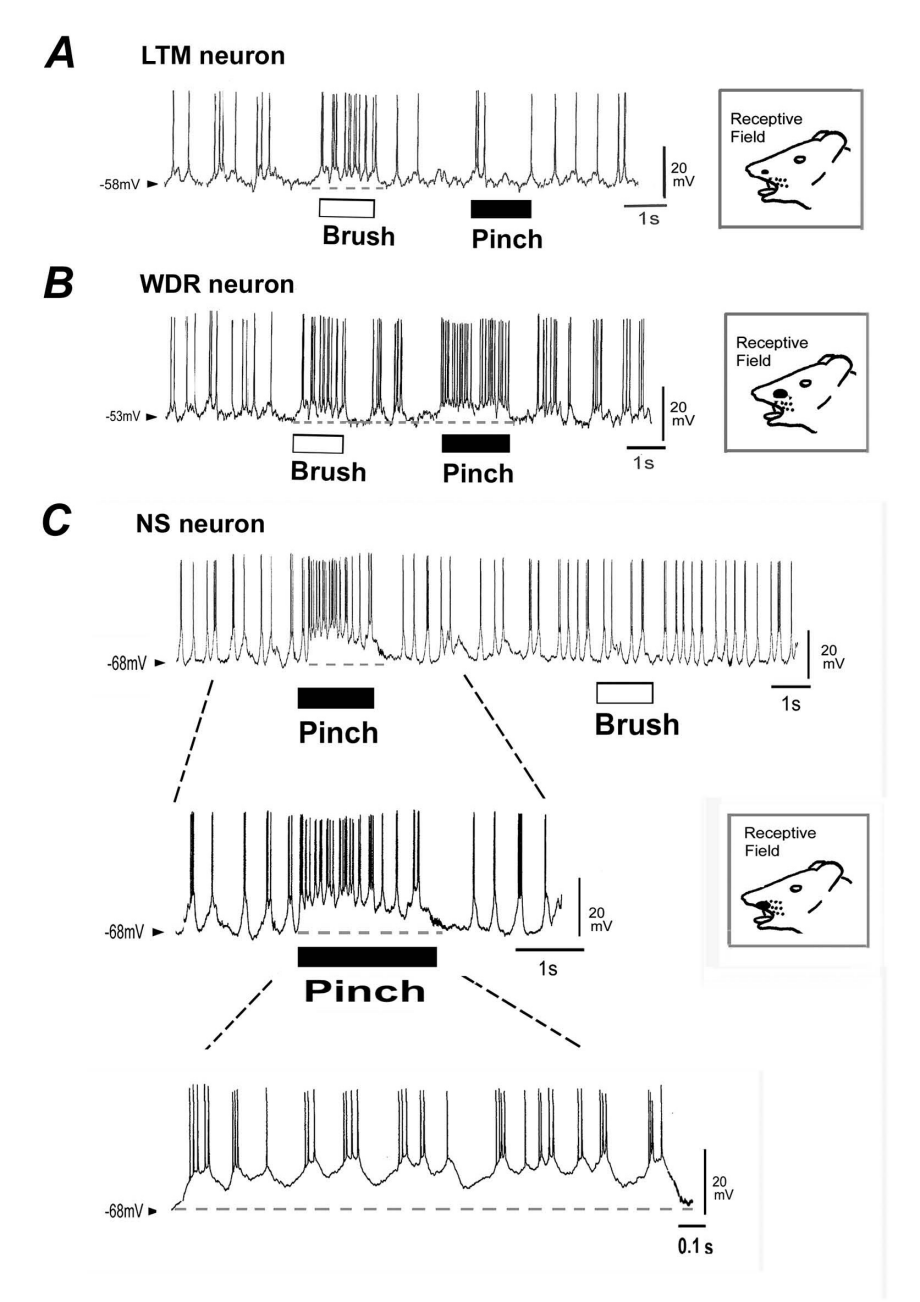
**Response properties of SI neurons responding to noxious mechanical stimulation**. ***A***: Low-threshold mechanoreceptive (LTM) neurons only responding to non-noxious stimulation (brush). ***B***: Example of wide-dynamic range (WDR) SI neurons responding to both noxious and non-noxious stimulation (blackened area). Arrow heads and broken horizontal line show resting membrane potential. ***C***: Example of nociceptive-specific (NS) neurons - noxious pinch stimuli applied to the orofacial skin (blackened area) produced a barrage of excitatory postsynaptic potentials (EPSPs) accompanied by action potentials in a SI neuron under current-clamp conditions. This neuron did not respond to non-noxious stimulation (brush). Arrow heads and broken horizontal line show resting membrane potential.

Twenty-seven out of 63 neurons were identified in the mechanical receptive field of the orofacial area (36 neurons showed no receptive field) and they were classified as both non-nociceptive and nociceptive neurons. A typical example of non-nociceptive neurons (low-threshold mechanoreceptive [LTM]; 6/27, 22%) is shown in Fig. 2A. These neurons responded to only non-noxious stimuli (brush), with weak membrane depolarization. The nociceptive neurons were further classified as WDR (3/27, 11%) or NS neurons (18/27, 67%); Fig. 2B shows a typical example of WDR neurons. Both noxious and non-noxious stimulation applied to the orofacial area evoked spikes discharges with membrane depolarization. These nociceptive neurons however were more sensitive to pinch stimuli than brush stimuli. Since the present study focused on the SI neurons encoding noxious stimulation, we examined the characterization of NS neurons responding to noxious stimulation of the orofacial area. A total of 15 SI neurons were successfully identified by biocytin injection. They were located at a depth of 750-900 μm, corresponding to layers III-V. Fig. 1B shows the location of cell body of each type of neurons. A typical morphological example of recorded NS neurons (pyramidal neuron: characteristic apical and basal dendritic trunks, 7/9, 78%) identified by biocytin is shown in Fig. 1C. While, a small number of NS neurons show morphological characteristics of non-pyramidal (multipolar) neurons (2/9, 22%). Also WDR neurons were identified in the pyramidal (2/3, 67%) and non-pyramidal (1/3, 33%) neurons. A typical example of noxious mechanical stimulation-responding SI neurons (NS neurons) is shown in Fig. 2C. Noxious pinch stimuli applied to the orofacial skin (blackened area) produced a barrage of EPSPs accompanied by action potentials in SI neurons under current-clamp conditions. The increased pinch-evoked discharge frequency was due to temporal summation of EPSPs (Fig. 2C). These neurons did not respond to non-noxious stimulation (brush) (Fig. 2C). The electrophysiological membrane properties (the resting membrane potential, input resistance and spontaneous background activity) of each type of neurons are summarized in Table 1. There is no significant difference of membrane properties among each types of neurons, as described in previous studies using the spinal dorsal horn neurons [35,36]. Although in this study, we found that both types of background firing, such as regular and burst firing in the LTM, WDR and NS neurons (for example, Fig 2A, regular firing; Figs. 2B, C, bursting firing), the majority of NS neurons (12/18, 67%) showed oscillatory bursting activity.

**Table 1:** Electrophysiological membrane properties of SI neurons.

Neuron type	No. of neurons	RMP (mV)	R_in _(MΩ)	Spontaneous activity	
				
				Firing pattern	Firing frequency (Hz)
LTM	6	-58.3 ± 3.5	34.1 ± 4.5	R (4), B (2)	2.6 ± 0.3
WDR	3	-55.1 ± 4.9	36.2 ± 3.1	R (1), B (2)	4.5 ± 1.2
NS	18	-61.5 ± 5.8	39.1 ± 5.2	R (6), B (12)	4.2 ± 0.8

LTM, Low- threshold mechanoreceptive; WDR, wide-dynamic range; NS, nociceptive specific. Resting membrane potential (RMP), the values reported refers to that taken during the hyperpolarized period (down-state) between ongoing spontaneous depolarization (up-state). The input resistance (R_in_) was calculated by injecting negative current into the soma and determing the voltage drop after current injection. Firing pattern of spontaneous activity: R, Regular pattern; B, bursting pattern. Values in parentheses are number of neurons.

### Response properties of nociceptive SI neurons responding to chemical stimulation applied to the receptive field

**Figure 3:**
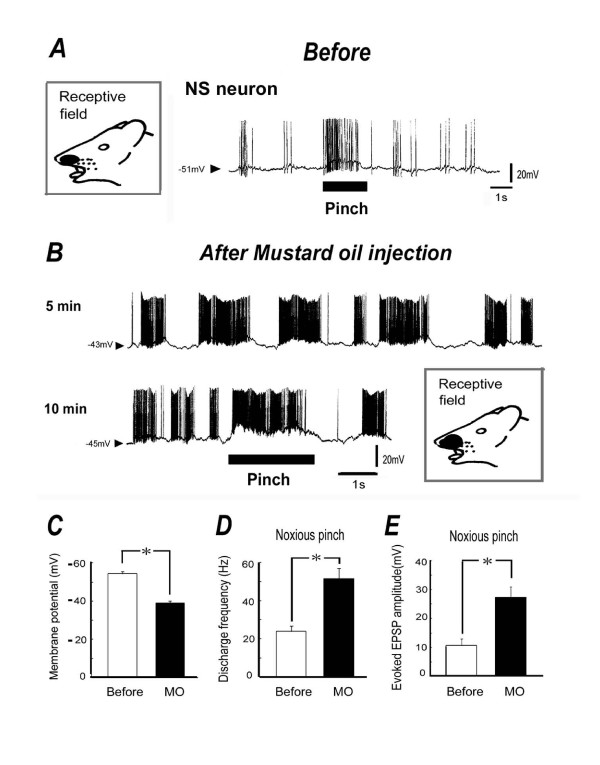
**Effect of noxious chemical stimulation of the receptive field on the spontaneous discharges and noxious pinch-evoked responses**. ***A***: Noxious pinch stimuli applied to the orofacial skin (blackened area) produced a barrage of excitatory postsynaptic potentials (EPSPs) accompanied by action potentials in SI neurons (NS-type). Blackened area indicates the location and size of the receptive field responding to noxious pinch stimulation. Arrow heads show resting membrane potential. ***B***: Example of responses of spontaneous discharges and of noxious pinch stimulation after subcutaneous injection of mustard oil (MO) into the receptive field area. Note that after MO injection (5 min), spontaneous discharges of SI neurons increased, lasting for 10-15 min. Noxious pinch-evoked discharge rate was increased and the response was accompanied by augmented EPSP amplitudes. ***C***: Change in the mean membrane potential of SI neurons after MO administration (10 min). *, *P *< 0.05. ***D***: Change in the mean noxious pinch evoked discharge of SI neurons after MO administration (10 min). *, *P *< 0.05. ***E***: Change in the mean noxious pinch evoked EPSP amplitude of SI neurons after MO administration (10 min). *, *P *< 0.05.

We next tested the effect of noxious chemical stimulation of the receptive field on both spontaneous and noxious pinch-evoked discharges. As shown in Fig. 3A, before application of MO (10 μl of 5%), noxious pinch stimuli applied to the orofacial skin produced a barrage of EPSPs accompanied by action potentials in SI neurons. After subcutaneous application of MO into the receptive field area, membrane potential shifted depolarization and spontaneous discharges of SI neurons gradually increased. An example of the spontaneous discharges and noxious pinch stimulation in response to MO application is shown in Fig. 3B. As shown in Fig. 3B, the SI neurons show clear oscillation which initiate bursting spike firings after MO application. After MO administration, the mean membrane potential in the SI neurons was significantly decreased (10 min, -53.3 ± 1.2 mV before MO vs. -39 ± 1.8 mV after MO, *P *< 0.05; Fig. 3C), and the mean spontaneous discharge frequency of the SI neurons was significantly increased (10 min, 5.3 ± 1.5 Hz before MO vs. 23.1 ± 2.2 Hz after MO, *P *< 0.05). In a similar fashion, the noxious pinch-evoked discharge rate was increased after MO application (10 min, 24 ± 2.9 Hz before MO vs. 52.1 ± 5.3 Hz after MO, *P *< 0.05; Fig. 3D), and was accompanied by augmented EPSP amplitudes. The mean EPSP amplitude and noxious pinch-evoked discharge frequency were significantly increased after MO application, compared to before application (10 min, 10.7 ± 2.3 mV before MO vs. 27.7 ± 5.3 mV after MO, *P *< 0.05; Fig. 3E). The size of the receptive field was expanded after MO injection (8.6 ± 2.3 mm^2 ^vs. 16.8 ± 3.4 mm^2^, n = 4, p < 0.05). The MO-induced changes in membrane potential, discharge rates and EPSP amplitude returned to control levels within 40-50 min.

## Discussion

### Methodological considerations

In this study, we examined the synaptic response properties of SI neurons to noxious stimulation applied to the orofacial area in urethane-anesthetized rats using *in vivo *patch-clamp analysis. For obtaining stable *in vivo *whole-cell recordings, we used a simple and effective method of applying collagenase locally over the dura at the recording site [26]. Since it is known that the dura acts as a physical barrier and keeps the integrity of the cerebral-spinal fluids and microenvironment surrounding the brain tissue, our approach using the retention of the dura mater provides significantly improved stability, as well as improved success in obtaining a high-quality seal. In addition, we performed a rapid perforated patch-clamp method, by using amphotericin B [31-33], to facilitate a rapid reduction in access resistance (for the whole-cell current-clamp recording). Indeed, we obtained the following results in this study: (1) All the SI neurons examined in this study had membrane potentials more negative than -50 mV; (2) spontaneous EPSPs and IPSPs were observed in most neurons recorded; and (3) the series resistance was between 20 and 50 MΩ. When considering all these observations, our *in vivo *patch-clamp recording techniques are valid for examining the synaptic response properties of SI neurons in urethane-anesthetized rats.

### Synaptic response properties of nociceptive SI neurons

It has been previously demonstrated that monkey and cat SI neurons in the deeper lamina encode the intensity of noxious thermal stimulation [5-9]. Rat SI cortical neurons also respond to noxious mechanical stimulation of the trigeminal receptive field and these neurons are classified as either NS or WDR neurons [10-12]. Recently, Peng et al. [37] reported that peripheral noxious stimulation in anesthetized rats produced an immediate electroencephalogram desynchronization resembling cortical arousal, while membrane potentials of SI neurons switched into a persistent depolarization state. In this study, we found that noxious pinch stimuli applied to the orofacial skin near the whisker produced a barrage of EPSPs accompanied by action potentials in SI neurons under current-clamp conditions. Most of the neurons recorded in this study showed that the characteristic of the pinch-evoked increase in discharge frequency was due to the temporal summation of EPSPs.

There is evidence that the primary sensory cortex responds to noxious stimuli, as revealed by imaging methods such as magnetoencephalography, positron emission tomography and functional magnetic resonance imaging [4]. Histological confirmation in our study showed that noxious pinch-responding neurons were located in the deeper layers III-V of the SI cortex. Majority of NS and WDR neurons had morphological characteristics indicative of pyramidal neurons, and some of these neurons were found in non-pyramidal neurons, as reported by previous studies [38,39]. This is also supported by evidence that NS neurons in the rat SI cortex are found almost exclusively in layers V and VI [40]. Since the location of nociceptive neurons is mainly concentrated in the deeper layer and these neurons do not respond to non-noxious stimulation, it can be assumed that functional pure-column nociceptive processing may not exist, in contrast to the tactile information processing system [6]. Actually, this is also supported by a human study showing that nociceptive processing apparently does not share the complex and hierarchical organization of tactile processing that is required by our elaborate sensory capacities [41].

Previously, Yoshimura et al. [42] reported that noxious pinch stimulation near a whisker pad did not produce any significant response in SI neurons located at 100 to 1000 μm from the surface of the cortex in urethane-anesthetized rats. In the present study, only 33% (21/63) of the SI neurons tested (at a depth of 750-900 μm) responded to noxious stimulation applied to the orofacial area. It has been shown that the spontaneous firing patterns and sensory responsiveness of the rat somatosensory cortex changes in a systematic way across progressive stages of urethane anesthesia [43]. Thus, the difference between our data and the previous report may be explained by a different depth of anesthesia. Indeed, there is evidence demonstrating that under light urethane anesthesia, SI cortical neurons are closely related to the spike threshold, exhibiting responsiveness to inputs from the thalamus and neighboring cortical columns [44]. Other factors also need to be considered - for example, the recording depth and stimulation site contribute to differences in SI neuron responses. However, further studies are needed to clarify these possibilities.

In this study, we also found a higher proportion of NS neurons than WDR neurons, as described in previous studies using several anesthetic agents (e.g., pentobarbital, ketamine, urethane and halothane) [5-12]. Although the precise reason for the difference between our data and previous reports is unclear, the difference may be explained by the different types of anesthetic agents and/or the depth of anesthesia [43-45]. Another possibility is the electrophysiological recording conditions, such as extracellular unit recordings vs.* in vivo *patch-clamp recordings.

### Functional significance of SI nociceptive neurons

MO is an agonist of the TRP ion channel family member, TRPA1 [13,18]. This agonist is known to activate somatosensory neurons, resulting in acute pain and neurogenic inflammation through the peripheral release of neuropeptides from the primary afferent nerve terminal [19]. Kerstein et al. [17] recently reported that acute pharmacological blockade of TRPA1 at the cutaneous receptive field inhibits formalin-evoked activation and markedly reduces mechanically-evoked C-fibre action potential firings. They concluded that functional TRPA1, at the level of the sensory afferent nerve terminals in the skin, plays an important role in the responsiveness to both noxious chemical and mechanical stimuli, including acute and chronic pain [17].

Woolf and King [20] reported that in the spinal cord, peripheral application of the chemical irritant MO produces a prolonged increase in the responses to low- and high-intensity mechanical stimuli applied to the receptive field and these effects are characterized by a depolarization response as well as increased amplitude of the EPSPs evoked by mechanical stimulation. In agreement with these findings, in the present study, after chemical stimulation of the mechanical receptive field of SI neurons, we observed the following findings: (1) the membrane potential gradually shifted depolarization; (2) the rates of spontaneous discharges gradually increased; and (3) noxious pinch-evoked discharge rates increased and the responses were associated with augmented EPSP amplitudes. Previous studies have reported a significantly increased pinch-evoked responsiveness for SI neurons after carrageenin-induced inflammation [25,46], which is in accordance with our findings. These results are quite similar to the responsiveness of neurons located in the ventrobasal nucleus of the thalamus [47]. Previous reports have also shown that the application of MO into several orofacial tissues induces hyperexcitability of the trigeminal spinal nucleus caudalis neurons and these changes contribute to the trigeminal hyperalgesia [21-24]. In this study, we also found that under urethane anesthesia the majolity of NS neurons (12/18, 67%) shows oscillatory bursting activity associated with thalamo-cortical activity [48] and these neurons show clear oscillation, which initiates bursting firing, after chemical stimulation of the mechanical receptive field. Recent human study shows that short-term sensitization of the esophagus resulted in central neuroplastic changes (e.g. evoked potential) involving the cingulate gyrus, which also showed pathological activation in functional diseases of gut, thus reflecting the importance of this region in visceral pain and hyperalgesia [49]. Our findings may indicate that the sensitization of peripheral receptor triggers an increase in the neuronal excitability of each relay station of neurons as well as SI neurons, and as a result, the increase of synaptic inputs of NS neurons in the SI cortex subsequently results in the central sensitization (neuroplastic changes) of NS neurons in the SI cortex. Taken together, these findings suggest that the excitability of SI cortical neurons are associated with trigeminal inflammatory hyperalgesia which may be due to both the mechanisms underlying peripheral and central sensitization. Our findings may have potential usefulness because treatment for acute or chronic pain includes mechanical hypersensitivity; however further studies are needed to elucidate this possibility.

## Conclusions

This is the first study to provide evidence that rat SI neurons in deep layers III-V respond to the temporal summation of EPSPs due to noxious mechanical and chemical stimulation applied to the orofacial area. These findings suggest that rat SI neurons in the deeper layers contribute to the processing of nociceptive information, including hyperalgesia.

## Abbreviations

SI: primary somatosensory; LTM: low-threshold mechanoreceptive; NS: nociceptive-specific; WDR: wide dynamic range; EPSPs: excitatory postsynaptic potentials; IPSPs: inhibitory post synaptic potentials; TRP: transient receptor potential; TRPA1: transient receptor potential ankyrin 1; MO: mustard oil; PBS: phosphate buffered saline;

## Competing interests

The authors declare that they have no competing interests.

## Authors' contributions

MT (C.A) participated in the design of the study, carried out all the experiments, analyzed the data and wrote the manuscript. MT helped with all experiments. MN assisted with histological identification of cells. SM provided data interpretation and helped to finalize the manuscript. All authors read and approved the final manuscript.
